# Pharmacological Modulation of Serotonin Levels in Zebrafish Larvae: Lessons for Identifying Environmental Neurotoxicants Targeting the Serotonergic System

**DOI:** 10.3390/toxics9060118

**Published:** 2021-05-25

**Authors:** Melissa Faria, Eva Prats, Marina Bellot, Cristian Gomez-Canela, Demetrio Raldúa

**Affiliations:** 1 Institute for Environmental Assessment and Water Research (IDAEA-CSIC), Jordi Girona 18, 08034 Barcelona, Spain; drpqam@cid.csic.es; 2 Research and Development Centre (CID-CSIC), Jordi Girona 18, 08034 Barcelona, Spain; eva.prats@cid.csic.es; 3 Department of Analytical Chemistry and Applied (Chromatography Section), School of Engineering, Institut Químic de Sarrià-Universitat Ramon Llull, Via Augusta 390, 08017 Barcelona, Spain; marina.bellot@iqs.url.edu (M.B.); cristian.gomez@iqs.url.edu (C.G.-C.)

**Keywords:** zebrafish larvae, behavior, serotonin, neurotransmitters, modulation

## Abstract

This study examines the effects of acute pharmacological modulation of the serotonergic system over zebrafish larvae’s cognitive, basic, and defense locomotor behaviors, using a medium to high throughput screening assay. Furthermore, the relationship between behavior, enzyme activity related to neurotransmitter metabolism, neurotransmitter levels, and gene expression was also determined. Modulation of larvae serotonergic system was accomplished by 24 h exposure to single and opposite pharmacodynamics co-exposure to three model psychopharmaceuticals with antagonistic and agonistic serotonin signaling properties: 2.5 mM 4-Chloro-DL-phenylalanine (PCPA) and 5 µM deprenyl and 0.5 µM fluoxetine, respectively. Similar behavioral outcome was observed for deprenyl and fluoxetine, which was reflected as hypolocomotion, decrease in larvae defensive responses, and cognitive impairment. Contrarily, PCPA induced hyperlocomotion and increase in larvae escape response. Deprenyl exposure effects were more pronounced at a lower level of organization than fluoxetine, with complete inhibition of monoamine oxidase (MAO) activity, dramatic increase of 5-HT and dopamine (DA) levels, and downregulation of serotonin synthesis and transporter genes. PCPA showed mainly effects over serotonin and dopamine’s main degradation metabolites. Finally, co-exposure between agonistic and antagonist serotonin signaling drugs reviled full recovery of zebrafish impaired locomotor and defense responses, 5-HT synthesis gene expression, and partial recovery of 5-HT levels. The findings of this study suggest that zebrafish larvae can be highly sensitive and a useful vertebrate model for short-term exposure to serotonin signaling changes.

## 1. Introduction

Animals are able to respond to changing environmental stimuli, such as food availability or predation, through different forms of behavioral plasticity, including arousal and associative and non-associative learning [1]. Any changes in normal behavioral conduct could have dramatic consequences from individual survival to ecological disaster. Behavioral plasticity is driven at the molecular level by the action of modulatory neurotransmitters [1,2]. Serotonin (5-hydroxytryptamine) is one of the major neurotransmitters in the central nervous system (CNS), modulating behaviors like mood, sleep, aggressiveness, fear, and appetite [3]. Correlation between abnormalities in the serotonergic system and several pathologies, including affective disorders, schizophrenia, and anxiety, has been demonstrated [3].

The rate-limiting step in the synthesis of serotonin is the conversion of tryptophan to 5-hydroxytryptophan (5-HTP) by the tryptophan hydroxylase (TPH). Serotonin is then synthesized from 5-HTP by the aromatic amino acid decarboxylase (AAAD), and is quickly transported from the cytoplasm to synaptic vesicles by the vesicular transporter SLC18A2 (VMAT2). When serotonin is released to the synaptic cleft via exocytosis, the serotonin transporter SLC6A4 (also known as SERT) is responsible for its uptake/reuptake to the serotonergic neurons. Serotonin is metabolized by MAO to 5-hydroxy-indolecetaldehyde which is quickly metabolized by an aldehyde dehydrogenase to form 5-hydroxyindoleacetic acid (5-HIAA), the major metabolite of serotonin [4].

Neuroactive chemicals, including both emerging and legacy pollutants, are the largest group of micropollutants present in European rivers [5]. The specific neurotoxic effect of environmental concentrations of some of these pollutants on different nontarget species, including fish, has been reported [6,7,8]. Considering the essential role played by serotonin in both neurotransmission and neuromodulation, neuroactive chemicals targeting serotonergic system of are of particular concern. Despite the fact that the effects of selective serotonin re-uptake inhibitors (SSRIs) in fish species have been extensively studied [9,10,11,12], information regarding other potential modes of actions, like TPH or monoamine oxidase (MAO) inhibition, is still scarce.

In this manuscript we have analyzed the basal locomotor activity, visual-motor response, and the vibrational startle response in 8 days post-fertilization (dpf) zebrafish larvae exposed during 24 h to neuroactive drugs specifically designed to increase (SERT inhibitor: fluoxetine; MAO inhibitor: deprenyl) or decrease (TPH inhibitor: chloro-DL-phenylalanine (PCPA)) serotonin levels. Moreover, changes in the profile of serotonergic and dopaminergic neurotransmitters in the heads of the exposed larvae, as well as changes in the expression of monoaminergic-related genes, have been assessed. 

## 2. Material and Methods

### 2.1. Fish Husbandry and Larvae Production

All procedures regarding fish housing, larvae production, and experiments were approved by the CID-CSIC’s Institutional Animal Care and Use Committees and carried out according to the institutional guidelines under a license from the local government (agreement n° 9027). For more detail please refer to Section S1.1. Fish Husbandry and Larvae Production, of the Supplementary Material.

### 2.2. Experimental Protocol

**Figure 1:**
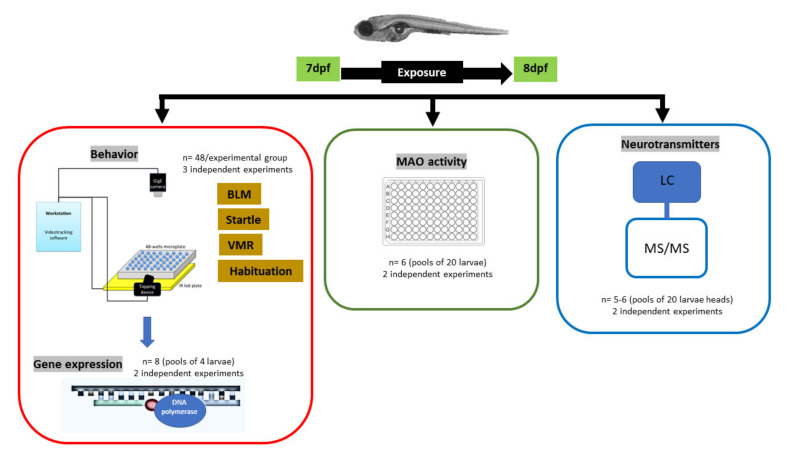
Diagram of the conducted study, indicating the exposure period (from 7 to 8 dpf) and the addressed variables (behavior, gene expression, MAO activity, and neurotransmitters), divided into their corresponding dataset (red, green, and blue squares). Indicated are also the number of larvae and independent experiments used for each variable.

All chemicals used were of certified laboratory high-quality grade purchased from Sigma-Aldrich (St. Louis, MO, USA): fluoxetine (CAS:56296-78-7); chloro-DL-phenylalanine (PCPA; CAS: 7424-00-2) and deprenyl (CAS:14611-52-0). Whereas stock solutions of deprenyl and fluoxetine were prepared in DMSO and then diluted 10^−3^ in fish water for exposure solutions, chloro-DL-phenylalanine (PCPA) exposure solution was prepared directly in fish water. Previous to the experiment, the selected compounds were evaluated for toxicity, which was established either by death, gross morphology and/or swimming impairment, or clear decrease in the escape response evoked by the tapping on the plate. The highest concentration, which did not induce any of the above-mentioned criteria, was selected for this study. DMSO was added to all exposure conditions to a final concentration of 0.1%. The use of 0.1% DMSO in vehicle controls has been reported to be safe and is commonly used in the screening of zebrafish libraries of small chemicals [13,14]. For behavior assessment, exposures were conducted in 48-well microplates containing 1 mL of exposure solution and 1 larva per well. Behavior trials were directly tested without further manipulation, following 24 h of exposure (larvae from 7 to 8 dpf) (Figure 1). For MAO activity and neurotransmitters assessment, exposures were carried out in 6-well plates, where each well contained 5 larvae and 5 mL of exposure medium, and treatments placed randomly across plates. This exposure window was chosen because by 7 dpf most of the central nervous system is quite well developed and the observed effects will be mainly related with neurotoxicity instead of developmental neurotoxicity. Moreover, by using this window, any major developmental effects of the selected chemicals is avoided, since larvae at this stage have already undergone most of their organogenesis (https://zfin.org/zf_info/zfbook/stages/, accessed on 7 May 2021), and a longer exposure was discharged to avoid having to feed larvae and therefore insert a new variable in the experiment. Exposures were performed at 28.5 °C (POL-EKO APARATURA Climatic chamber KK350, Poland) with 12L:12D photoperiod. Before sampling, larvae were euthanized by rapid chilling, transferring the larvae to ice-chilled water (2–4 °C), which is a method of euthanasia in accordance with the AVMA Guidelines on Euthanasia (https://www.avma.org/resources-tools/avma-policies/avma-guidelines-euthanasia-animals, accessed on 13 May 2021). Samples were transferred into an Eppendorf Tube and all medium water was removed. They were then immediately frozen with dry ice and then stored at −80 °C until further analysis. Larvae used for behavioral trials were then sampled for qRT-PCR analysis (*n* = 4/pool) while due to the large number of larvae required for neurotransmitters and MAO activity (*n* = 20/pool) assessment experiments were conducted separately (Figure 1). For each variable investigated, larvae were collected from 2–3 trials of the same experiment setup conducted in different days and with different batches of animals (Figure 1).

### 2.3. Behavioral Analysis

Vibrational startle response assay was performed as described in [8]. The basis of this test is the escape response evoked in zebrafish larvae by a tapping stimulus. Video tracking was acquired, and EthoVision XT 9 software (Noldus, Wageningen, The Netherlands) was used to analyze the escape response. Trials were performed at 28 °C with near-infrared light. The highest intensity (intensity level: 8) was selected for the tapping stimulus and, after a 15 min acclimation period to the chamber, 50 stimulus were delivered, one every second. Videos were recorded at 30 frames per second and the vibrational startle response (VSR) for each individual larva was analyzed by measuring the distance traveled (cm) over the 1 s period following each stimulus. “Startle Response or Startle” is defined as the total distance moved (cm) in response to the first stimulus and “Habituation or non-associative learning” as the area under the curve (AUC) of plots of distance moved relative to the response to the first stimulus [8].

Basal locomotor activity (BLM) and visual-motor response (VMR) analyses of 8 dpf zebrafish larvae were conducted with the DanioVision system associated with the Ethovision XT 11 software (Noldus, Wageningen, the Netherlands), as described by [15]. Before video recording, larvae were first acclimated for 20 min under dark conditions. Video tracking trials consisted of a 40 min cycle with a 15 min dark period followed by a 10 min light period followed by a 15 min of darkness. The BLM activity is classified as the total distance (cm) traveled by larvae during the last 10 min of the first dark cycle. The VMR is based in the hyperactivity period induced by a sudden absence of light [16], represented as the difference between total distance (cm) traveled for two minutes after and before to the beginning of the light cycle.

### 2.4. RNA Preparation and qRT-PCR Analysis

Analysis of larvae gene expression was conducted as previously described by Prats et al., 2017. Total RNA was extracted from 6–8 pools of 4 larvae (8 dpf), collected from two independent experiments, using the Trizol Reagent (Invitrogen Life Technologies, Carlsbad, CA). Concentration of RNA was measured in a NanoDrop™ ND-8000 spectrophotometer (260 nm) (Fisher Scientific) and its quality was checked using an Agilent 2100 Bioanalyzer (Agilent Technologies, Santa Clara, CA, USA). Values of RNA Integrity Number (RIN) ranged between 9 and 10. Following DNaseI treatment (Ambion, Austin, TX, USA), 1 µg of RNA was employed to synthesize the first strand of complementary (cDNA) using the First Strand cDNA synthesis Kit (Roche Diagnostics, Germany) and oligo(dT), according to the instructions provided by the manufacturer.

Real Time PCR was performed in a LightCycler^®^ 480 Real-Time PCR System with SYBR Green PCR Master Mix (Roche Diagnostics, Mannheim, Germany). Cycling parameters were 15 min at 95 °C followed by 45 cycles of 10 s at 95 °C and 30 s at 60 °C. For each experimental condition, qPCR analyses were performed with three technical replicates for each sample. Primer sequences (Sigma-Aldrich, Steinheim, Germany) of the four selected genes (*tph1a*, *mao*, *sert,* and *vmat2*) are reported in Supplementary Table S1. Previous to any analysis, all primers were checked for their efficiency and specificity. Results were normalized using the housekeeping *ppia2* as reference gene [17] and the relative abundance of mRNA was calculated following the ΔΔCt method [18] deriving fold-change ratios from these values. The housekeeping gene remained stable across all treatments (Supplementary Table S4).

### 2.5. Zebrafish Monoamine-Oxidase (MAO) Activity

8 dpf zebrafish larvae were collected in pools of 20 individuals and homogenized in ice-cold 10 mM Phosphate Buffer pH 7.6 with 1 mM EDTA, to a final tissue volume concentration of 100 larvae/mL of buffer using a TissueLyser^®^ (Qiagen, Germantown, MA, USA). After centrifuging homogenates at 2500× *g* for 5 min at 4 °C, MAO activity was immediately determined in the supernatant using the peroxidase-linked spectrophotometric assay described by Holt et al. [19] and adapted to zebrafish tissue by [20], based on the determination of the amount of H_2_O_2_ released during the oxidation of amines. For further information please refer to Supplementary Material Section S1.2. Zebrafish monoamine-oxidase (MAO) activity.

### 2.6. Extraction and Analysis of Neurotransmitters

Monoaminergic neurotransmitters were extracted from 8 pools of 20 larvae heads according to the procedure adapted from Mayol-Cabré et al. [21] based on the use of a solvent of similar polarity to that of the neurotransmitters in order to be extracted from the sample. Ultra-high-performance liquid chromatography (Acquity UPLCH-Class Waters, Milford, MA, USA) coupled to a triple quadrupole mass spectrometer equipped with an electrospray (ESI) source (Xevo, TQS micro, Waters, Milford, MA, USA) was used to perform the analysis. Additional details on the extraction and analysis of neurotransmitters are provided in the Supplementary Material, Section S1.3. Monoaminergic neurotransmitters extraction and analysis.

### 2.7. Statistical Analysis

Analysis of data was performed with IBM SPSS v25 (Statistical Package 2010, Chicago, IL, USA) and plotted with GraphPad Prism 8.31 for Windows (GraphPad software Inc, La Jolla, CA, USA). Data normality was assessed using Kolmogorov–Smirnov and Shapiro–Wilk tests. Multiple comparison tests were used to determine differences between normally distributed groups, while the Kruskal–Wallis test followed by Dunn’s multiple comparison test against the control value was applied to test for differences between groups that did not meet parametric assumptions. One-way ANOVA followed by either Dunnett’s or Tukey’s test was used to compare results with those of the control group, or to determine homogenous subset groups, respectively. Scattered plots of data are presented in figures, with the median depicted as a red line. Significance was set at *p* <  0.05.

## 3. Results

### 3.1. Serotonergic Modulation—Response at the Organismal Level

**Figure 2:**
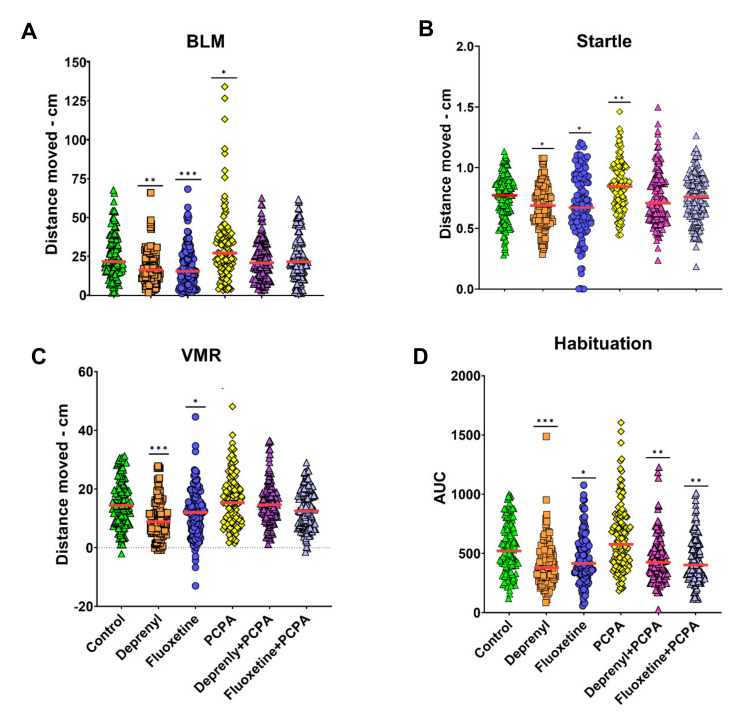
Behavioral changes on zebrafish 8 dpf old larvae, following 24 h waterborne exposure to 5 µM Deprenyl, 0.5 µM Fluoxetine, 2.5 mM PCPA, and the combination of 2.5 mM PCPA with either Deprenyl 5 µM or Fluoxetine 0.5 µM. (**A**) Basal locomotor (BLM) activity, represented as the total distance (cm) travelled during 10 min (*n* = 126–132); (**B**) acoustic/vibrational escape response (startle), represented as the total distance (cm) travelled following the delivery of a tapping stimulus (*n* = 127–133); (**C**) visual-motor response (VMR), representing the response of larvae due to transition of light to dark, represented as the difference of the total distance (cm) travelled by larvae during two minutes after and before the transition of light to dark (*n* = 121–126); (**D**) habituation of the acoustic/vibrational escape response evoked by a series of 50 tapping stimulus delivered every second represented as area under the curve (AUC) of larvae responses (*n* = 124–132). Data are from 3 independent experiments and are reported as scatter plots with the median (red line). Significance was set to *p* < 0.05 and can be represented as * when *p* < 0.05; ** when *p* < 0.01 and *** when *p* < 0.001, Kruskal–Wallis non-parametric test.

A battery of behavioral tests including basal locomotor (BLM) activity, visual motor response (VMR), the acoustic/vibrational escape response (also referred as startle response) and the habituation of the startle response were used to assess the behavioral profile in zebrafish larvae exposed to prototypic compounds inhibiting MAO (deprenyl), SERT (fluoxetine), and TPH (PCPA) activities. The selected concentrations were the same as those used in a previous study [22], and similar to the latter study, no system toxicity or morphological effects were observed in larvae following exposure. A significant effect in larvae BLM was found in those exposed to deprenyl, fluoxetine, and PCPA (Figure 2A) (*H*(5) = 37.310, *p* = 0.000). Whereas, deprenyl and fluoxetine significantly decreased larvae BLM (respectively, *p* = 0.000 and *p* = 0.003), the opposite was observed for PCPA (*p* = 0.027). Furthermore, the two serotonin level enhancer drugs showed a more prominent effect than its counter drug. Curiously, complete recovery of this behavior was observed in both combined exposure conditions (Figure 2A). The same behavior was observed for the startle response of zebrafish larvae (*H*(5) = 42.889, *p* = 0.000) (Figure 2B), however, the effects in this behavior where slightly milder compared to the BLM. Full recovery of the impaired escape response was also observed when combining PCPA with either deprenyl or fluoxetine (Figure 2B). The VMR was only impaired by deprenyl and fluoxetine (*H*(5) = 53.180, *p* = 0.000), with deprenyl presenting a stronger effect over this response (Figure 2C). In spite of this, similar to the previous two behavioral responses, a full recovery was observed when in combination with PCPA. Habituation of the escape response was the only behavioral response with a more distinct outcome (Figure 2D). The non-associative learning profile was impaired by deprenyl and fluoxetine alone and in combination with PCPA (*H*(5) = 37.316, *p* = 0.000), with deprenyl, the MAO activity inhibitor, once more, presenting stronger effects than the SSRI, fluoxetine. 

### 3.2. Serotonergic Modulation—Response at the Molecular Level

In order to better understand the changes observed in larvae behavioral outcome, following 24 h exposure to known drugs that increment and decrease serotonin levels and their combination, the neurotransmitter, biochemical and gene expression profiles were addressed. 

#### 3.2.1. Neurotransmitter Profile

**Figure 3:**
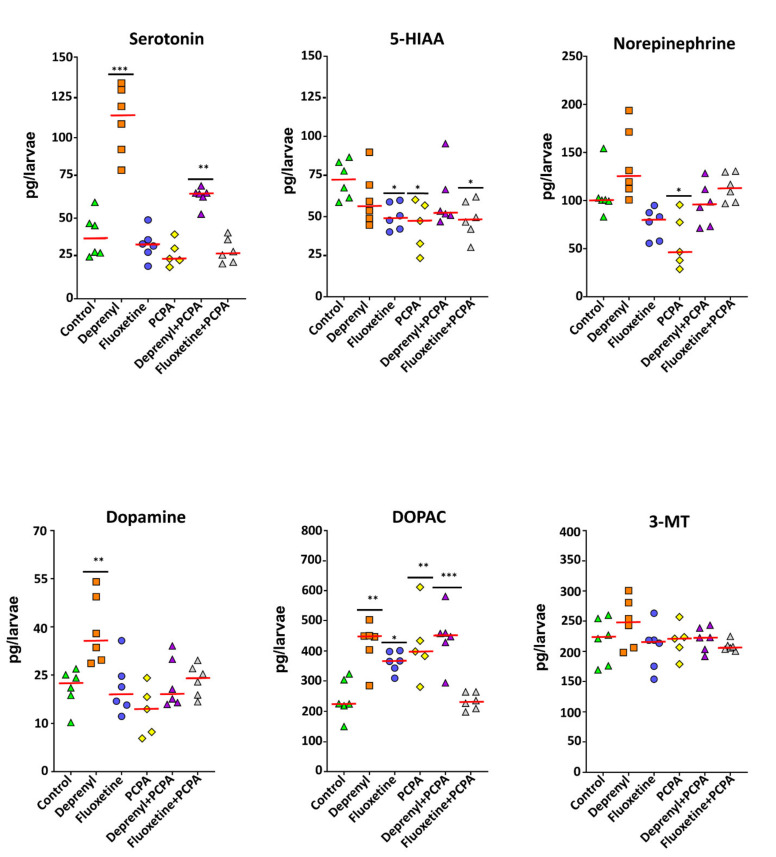
Monoaminergic neurotransmitter profiles of heads of zebrafish exposed to 5 µM Deprenyl, 0.5 µM Fluoxetine, 2.5 mM PCPA, and the combination of 2.5 mM PCPA with either Deprenyl 5 µM or Fluoxetine 0.5 µM. Data are reported as scatter plots with the median (red line) and *n* = 5–6 for all treatment groups. Significance was set to *p* < 0.05 and can be represented as * when *p* < 0.05; ** when *p* < 0.01 and *** when *p* < 0.001, one-way ANOVA with Dunnett’s multiple comparison test.

The profile of the monoaminergic neurotransmitters serotonin (5-HT) and dopamine (DA), as well as their products, norepinephrine (NE), 5-hydroxyindoleacetic acid (5-HIAA), 3,4-dihydroxyphenylacetic acid (DOPAC), and 3-methoxytyramine (3-MT) were determined in the heads of control and exposed larvae (Figure 3). Levels of all neurochemicals with the exception of 3-MT were significantly affected by the treatments (*p* < 0.05, Supplementary Table S2—One-way ANOVA results of monoaminergic neurotransmitter levels). In a single exposure scenario of the selected drugs, deprenyl triggered a strong increase of serotonin levels (*p* < 0.000, Dunnett’s test). The remaining drugs did not affect levels of this neurotransmitter. On the other hand, an important recovery of serotonin levels could be observed when deprenyl was combined with PCPA (Supplementary Table S3), however, the observed recovery was not able to reach similar levels to those of unexposed larvae (*p* = 0.007, Dunnett’s test) (Figure 3). Levels of the degradation product of serotonin, 5-HIAA was significantly deterred by fluoxetine (*p* = 0.034, Dunnett’s test) and PCPA (*p* = 0.010, Dunnett’s test), with the latter having a stronger effect over 5-HIAA levels (Figure 3, Supplementary Table S3). In addition, this same trend could be observed in larvae exposed to the combination of fluoxetine + PCPA (*p* = 0.022, Dunnett’s test), however PCPA’s strong effect seemed to have been slightly attenuated by the presence of fluoxetine (Figure 3, Supplementary Table S3). Similar to serotonin, dopamine levels were also significantly increased by deprenyl (*p* = 0.002, Dunnett’s test), which were then fully recovered when combined with PCPA (Figure 3, Supplementary Table S3). Furthermore, levels of the MAO-mediated degradation product of dopamine, DOPAC, were found significantly higher than those of control following exposure to deprenyl and curiously also fluoxetine and PCPA (*p* = 0.001, *p* = 0.03, and *p* = 0.002, Dunnett’s test, respectively). Furthermore, in combined exposure setups, that of deprenyl + PCPA was unable to recover DOPAC levels while full recovery could be observed for fluoxetine + PCPA (Figure 3, Supplementary Table S3). Finally, levels of norepinephrine, a neurotransmitter synthesized from dopamine through dopamine β-monooxygenase activity, was significantly decreased by PCPA (*p* = 0.010, Dunnett’s test) (Figure 3), which then recovered when PCPA was combined with either deprenyl or fluoxetine (Supplementary Table S3).

#### 3.2.2. Gene Expression and MAO Activity

**Figure 4:**
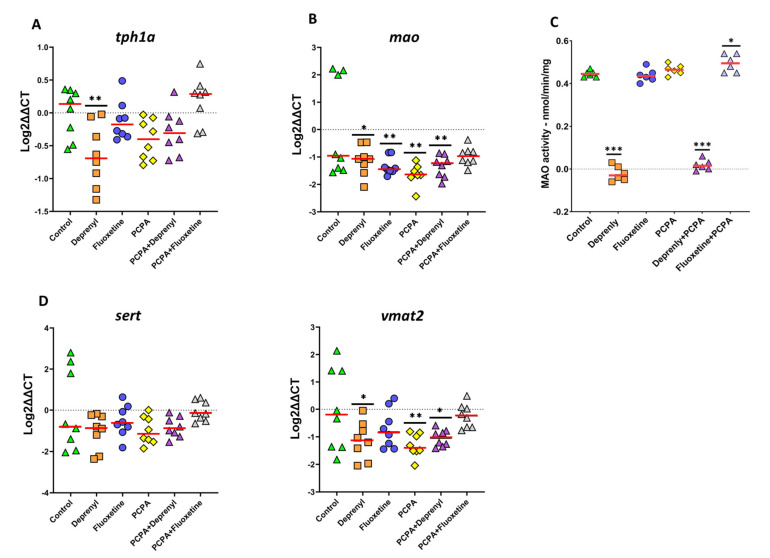
Effects over gene and enzyme activity levels in whole body of 8 dpf old zebrafish larvae following pharmacological modulation of the serotonergic system. (**A**) Expression of *tph1α* involved in serotonin synthesis process; (**B**,**C**) Expression of *mao* and MAO activity, respectively, involved in the degradation of serotonin; (**D**) Expression of *sert* and *vmat2* genes that respectively regulate serotonin transport from the synaptic cleft back to the presynaptic neuron and the transport of serotonin form the cell cortisol into synaptic vesicles. Data are from 2 independent experiments and are reported as scatter plots with the median (red line) and *n* = 6–8 for all treatment groups. Significance was set to *p* < 0.05 and can be represented as * when *p* < 0.05; ** when *p* < 0.01 and *** when *p* < 0.001, one-way ANOVA with Dunnett’s multiple comparison test.

The expression of genes involved in the 5-HT synthesis (*tph1a*), transport (*sert* and *vmat2*), and degradation (*mao*) as well as the functional activity of MAO was determined in the whole body of control and exposed larvae (Figure 4). Analysis of variance between groups for *tph1a* expression, gene encoding the rate limiting enzyme for serotonin synthesis, showed significant differences (F_5,42_ = 5.458, *p* = 0.001), which were mainly observed due to the strong downregulation of its expression in zebrafish larvae following 24 h of exposure to 5 µM of deprenyl (*p* = 0.004, Dunnett’s test) (Figure 4A). The remaining treatments of single exposures showed mild effects over *tph1a* expression but none were significantly different from control. On the other hand, the effect induced by deprenyl exposure was mostly recovered by combination exposure with PCPA (Figure 4A, Supplementary Table S4). The expression of the *mao* gene was significantly downregulated by most of the treatments (F_5,42_ = 3.934, *p* = 0.005) (Figure 4B). In single exposures, the strongest effect was observed for PCPA (*p* = 0.001, Dunnett’s test) followed by fluoxetine and deprenyl (*p* = 0.009 and 0.034, Dunnett’s test, respectively). In combination exposures, whereas deprenyl + PCPA failed to recover expression levels (*p* = 0.009, Dunnett’s test), fluoxetine + PCPA was able to rescue *mao* gene expression to similar levels as those expressed in control larvae. Next, to better understand MAO’s potential role in the observed changes, zebrafish MAO activity was determined. Despite the observed mild effects in *mao* expression in the presence of deprenyl, MAO activity was completely abolished by deprenyl (*p* < 0.001, Dunnett’s test) (Figure 4C, Supplementary Table S4). Furthermore, the presence of PCPA was unable to shift deprenyl’s dramatic effect over MAO’s activity. Curiously, the combined exposure of fluoxetine with PCPA significantly incremented the activity of this enzyme (Figure 4C, Supplementary Table S4). Finally, of the two investigated serotonin transporter genes, only *vmat2* expression was affected by the treatments (F_5,42_ = 3.581, *p* = 0.009). Both deprenyl and PCPA significantly downregulated the expression of this gene (*p* = 0.026 and 0.007, Dunnett’s test, respectively). Downregulated *vmat2* expression levels by PCPA were then recovered when co-exposed with fluoxetine (*p* > 0.05) (Figure 4D, Supplementary Table S4), while deprenyl only offered a slender recovery which was not enough to reach similar expression levels as those found in control larvae (*p* = 0.043) (Figure 4D).

A review of the main results can be found in Supplementary Table S5.

## 4. Discussion

There is an increased concern for the presence of neuroactive compounds targeting the serotonergic system in many aquatic ecosystems, as changes in serotonin levels may impair many behaviors essential for population survival. The mode of action of these compounds, including both legacy and emerging pollutants, includes inhibition of serotonin synthesis, re-uptake, and degradation [23,24,25,26].

Considering that zebrafish is one of the animal models more widely used in ecotoxicology, in this study we have characterized the behavioral effect in larvae of this species of three prototypic modulators of this neurotransmitter system: deprenyl (MAOB inhibitor), fluoxetine (selective serotonin re-uptake inhibitor, SSRI), and PCPA (tryptophan hydroxylase inhibitor).

### 4.1. Increase of Serotonin Signaling

Serotonin plays a fundamental role in modulating multiple brain functions and motor pathways in vertebrates. The zebrafish’s serotonergic system shares similarities with the respective mammalian systems, which makes this species a feasible model for evaluating its general properties. Here, behavioral modulatory effects of deprenyl and fluoxetine were evaluated at genetic, protein, and neurochemical levels. The overall results showed that 24 h exposure to 5 µM deprenyl had a potent effect over larvae serotonergic pathways.

A total inhibition of zebrafish MAO activity has been found in larvae exposed to 5 µM deprenyl for only 24 h. As expected, fluoxetine, a chemical targeting SERT activity, did not affect the activity of this enzyme. A similar inhibition of MAO activity by deprenyl was recently reported in zebrafish larvae [27]. Furthermore, Sallinen at al. (2009) also found that exposure to deprenyl strongly decreased zebrafish MAO activity in 7 dpf larvae [26]. However, in spite of using higher deprenyl concentrations (100 µM vs. 5 µM) and longer exposure times (7 days vs. 1 day), the final effect of deprenyl MAO activity of the larvae was stronger in the present study (100% vs. 74%). The observed discrepancies may most likely be due to differences in the exposure conditions (developmental exposure vs. short term larval exposure) or in the strain of zebrafish used. Curiously, at the gene expression level, downregulation of the *mao* gene was observed for both deprenyl and fluoxetine. In contrast, no effect over zebrafish *mao* expression was detected following 79 h exposure to 0.5 µM fluoxetine [28]. However, it is worth mentioning that the exposure period in this study was between 1 to 80 h post fertilization (hpf).

The inhibition of MAO activity by deprenyl led to a significant increase in the serotonin (about 300% of the control values) and dopamine (about 150% of the control values) levels in the head of the treated larvae, as well as the downregulation of expression levels of *tph1a* and *vmat2* genes, which encode for tryptophan 5-monooxygenase the rate-limiting enzyme for serotonin synthesis and for the vesicular monoamine membrane transporter, responsible for serotonin transport from the cellular cytosol into the synaptic vesicles. These results are consistent with the fact that zebrafish MAO displays a stronger affinity for serotonin than for dopamine [20]. Furthermore, they are also within the same line as those reported by Sallinen et al. (2009), in which MAO inhibitory activity by deprenyl exposure was accompanied by a high increase in serotonin levels (up to 169% of control values after 0–5 dpf treatment with 100 μM, and up to 977% of control values after 0–7 dpf treatment with 100 μM) [26]. Interestingly, although abolition of MAO activity strongly increased serotonin, 5-HIAA levels remained unchanged in the head of deprenyl- treated larvae. This result contrasts with the dramatic decrease in the 5-HIAA levels reported in larvae treated with 100 µM deprenyl from 0–5 dpf [26]. However, different studies on the effect of MAO inhibitors performed on rodents also show increased serotonin without changes in 5-HIAA levels [29,30]. In contrast to deprenyl, fluoxetine did not affect total serotonin levels in the heads of zebrafish larvae. Acutely, SSRIs, such as fluoxetine, are designed to increase synaptic availability of serotonin by blocking the pre-synaptic serotonin transporter (SERT) and preventing its re-uptake into the pre-synaptic terminals, which does not necessarily reflect changes of total central serotonin levels [31]. However, no effect over zebrafish *sert* transcript was detected following 24 h exposure to 0.5 µM fluoxetine. Other studies, such as Airhart et al. (2007) and Cunha et al. (2018) have indeed reported a downregulating effect of fluoxetine over zebrafish *sert* transcript levels following acute exposure to 4.6 µM from 4–5 dpf or exposure to 0.5 µM from 1–80 hpf, respectively. Then again, discrepancies in exposure conditions complicate result correlations [28,32].

One of the first observations on the role of the serotonergic system in mammalian behavior concerns arousal, which usually manifests as locomotion impairment. In this study, we first studied the effect of deprenyl and fluoxetine over the motor function of the larvae, where a significant decrease in the basal locomotor activity was found, a result consistent with the decreased locomotor activity reported in larvae exposed for 2 h to 100 µM deprenyl at 7 dpf [26] and 24 h to 4.6 µM fluoxetine (4–5 dpf) [32]. The same response pattern was observed when the arousal state of larvae was addressed by triggering sensory responses following visual and vibrational stimuli. Both compounds exhibited a significant decrease in the magnitude of the escape response evoked by either stimulus. Whereas the effect on the vibrational startle was consistent with that reported in a previous study [8], this is the first evidence using the visual-motor response paradigm. As MAOB loss of function may lead to decreased anxiety-like responses in rodents [33], the reduced response to aversive stimuli found in deprenyl-treated larvae may be considered as an anxiolytic-like effect of the hyperserotonergic phenotype. In a similar way, the prototypic SERT inhibitor fluoxetine also decreased 7 dpf zebrafish larvae escape response in the bouncing ball assay following acute exposure [34]. Non-associative learning has been studied in zebrafish larvae by monitoring the reduction in a startle response to a series of acoustic or vibrational stimuli [22,35]. Similar to this study and under the same exposure conditions, in a previous study, both compounds impaired zebrafish larvae by rapid decrease of larvae movement following consecutive tapping stimuli [22]. It has been reported that serotonergic neurons in addition to the Mauthner cells play an important role in the regulation of this form of learning in zebrafish; for example, Pantoja et al. (2016) reported decrease of total distance moved by larvae under habituation conditions following the treatment with quipazine, a serotonin receptor agonist [36].

### 4.2. Decrease of Serotonin Signaling

Exposure to the tryptophan hydroxylase inhibitor PCPA to reduce serotonin synthesis was used to investigate the impact of serotonin depletion in zebrafish larval locomotor behavior, escape responses, and learning. We examined the effects of serotonin reduction on the expression of mRNA transcripts, levels of neurochemicals, and enzyme activity associated with serotonin action.

As expected, PCPA did not affect zebrafish MAO activity following 24 h exposures to 2.5 mM. However, at the transcript level, *mao* expression was downregulated in exposed larvae. A possible explanation could be that underlying homeostatic mechanisms can be activated in response to changes in serotonergic signaling, such as decrease in serotonin stores. In this study, larvae exposed to 2.5 mM PCPA presented the lowest level of 5-HT across all studied compounds, with a decrease of 29% relative to control. Despite this, it was not found significantly different from larvae control levels; however, it could have been sufficient to activate adaptive mechanisms. This can be also observed in the downregulation of *vmat2* expression. The vesicular monoamine transporter type 2 (VMAT2) has an essential role in the storage and synaptic release of all monoamines, including serotonin. A two-way regulation mechanism between the activity of this monoamine transporter and levels of monoamines has already been reported [37,38]. Whereas the observed decrease of 5-HT levels was not significant from control, low levels of 5-HIAA suggests a decrease in serotonin synthesis.

Behavioral evaluation of PCPA-exposed larvae induced opposite effects of those observed for deprenyl and fluoxetine. Larvae exhibited hyperlocomotion (increased BLM), which is consistent with anxiety-like behaviors [39] and an increase in the escape behavior following a vibrational stimulus. Data about the behavioral effect of PCPA in zebrafish are scarce, and those found are mainly focused on developmental approaches, with controversial results [26,40]. On the other hand, PCPA increased rat behavioral response to turning off the electrical stimulation of the dorsal periaqueductal gray or to acoustic stimulus [41,42]. Analogous to this study, other studies have demonstrated that depleted levels of serotonin in mice have been associated to increased performance of escape-like behaviors [38,43].

### 4.3. Modulation of Serotoning Signaling

In order to determine if there was a direct relationship between serotonin and the behavioral effects induced by deprenyl and fluoxetine, co-exposure experiments of these chemicals with PCPA were conducted. We observed a partial but significant recovery of the normal serotonin levels in deprenyl + PCPA exposure along with full recovery of *tph1a* expression. Furthermore, total recovery of the BLM activity and VMR and vibrational startle response for all combined exposures was found, suggesting that serotonin may be a key modulator of these behaviors in zebrafish larvae. Re-establishment of the serotonin levels and partial improvement in the locomotor activity has been previously reported in larvae co-treated with 100 µM deprenyl and 1.5 mM PCPA [26]. These results strongly suggest that serotonin is directly involved in the observed effects of deprenyl and fluoxetine on larvae behaviors. Interestingly, treatment with PCPA resulted also in a full recovery of the dopamine levels, a result consistent with the reported lack of selectivity for TPH over tyrosine hydroxylase (TH), exhibited by PCPA when this chemical is used at high concentrations [44]. Therefore, it is not possible to discard a contribution of dopamine in the observed behavioral effects.

## 5. Conclusions

As a final remark, our results show that zebrafish larvae can be highly sensitive to prompt serotonin signaling changes, which further reinforces the use of this model vertebrate addressing behavioral and physiological roles of serotonin. Prototypic serotonin modulator chemicals able to decline or enhance serotonin signaling lead to opposite behavioral outcomes in zebrafish larvae following 24 h of exposure. Furthermore, behaviors were then recovered in combined exposure of chemicals with opposed modes of action. In addition, modulation of the larvae serotonergic pathway was also observed at lower levels of biological origination. A review of the obtained results is available in the Supplementary Material Table S5.

The findings presented in this study can provide a useful lesson for quickly identifying the presence of serotonin modulators in the environment: (1) an environmental sample presenting decrease in all four of the studied behaviors (the observed effect will be consistent with a serotonergic-like phenotype (MAO inhibition or SSRI)); (2) analysis of levels of monoaminergic neurochemicals, especially serotonin, in larvae heads; (3) measurements of MAO activity to confirm or discharge if the chemicals’ mode of action is through inhibition of serotonin metabolization or reuptake.

## Data Availability

Data supporting reported results will be provided upon reader’s request.

## Supplementary Materials

The following are available online at https://www.mdpi.com/article/10.3390/toxics9060118/s1, Supplementary Methods and Supplementary Results—Tables and the Animal Research: Reporting of Experiments (ARRIVE) guidelines checklist declaration. Table S1: List of primers used for the qPCR. Table S2: One-way ANOVA results of monoaminergic neurotransmitter levels. Table S3: Homogeneous Subsets for neurotransmitter levels following Tukey (HDS) post hoc analysis. Table S4: Homogeneous Subsets for gene expression results following Tukey (HDS) post hoc analysis. For *ppia2* CP (crossing point) values were used and ∆∆CT values were used for the remaining genes. Table S5: Review of main observed results of this study. Arrows pointing up or down indicate significant increase or decrease of responses, respectively. Absence of responses are indicated by a hyphen. Table S6: Animal Research: Reporting of Experiments (ARRIVE) guidelines checklist (doi:10.1371/journal.pbio.3000411).
