# Supplementary material for: Pharmacological Modulation of Serotonin Levels in Zebrafish Larvae: Lessons for Identifying Environmental Neurotoxicants Targeting the Serotonergic System
Source: Toxics. 2021 May 25;9(6):118. doi: 10.3390/toxics9060118 (PMC8227033; doi:10.3390/toxics9060118)
Supplement: Supplementary file 1 [file toxics-09-00118-s001.zip › toxics-1205829-supplementary.pdf]

Supplementary Material: Pharmacological Modulation of Serotonin Levels in Zebrafish Larvae: Lessons for Identifying Environmental Neurotoxicants Targeting the Serotonergic System

Melissa Faria, Eva Prats, Marina Bellot, Cristian Gomez-Canela, Demetrio Raldúa

1. Supplementary Methods

1.1. Fish Husbandry and Larvae Production

Adult wild-type zebrafish were purchased from EXOPET (Madrid, Spain) and maintained in fish water [reverse-osmosis purified water containing 90 µg/mL of Instant Ocean (Aquarium Systems, Sarrebourg, France) and 0.58 mM $CaSO4·2H2O$] at 28 ± 1 °C in the Research and Development Center of the Spanish Research Council (CID-CSIC) facilities under standard conditions. Embryos were obtained by in-tank group breeding with a 5:3, female:male ratio per tank. Breeding tanks are homemade and include a solid external tank and an internal plastic net. Embryos deposited in the bottom of the tank were collected and maintained in 500 mL glass containers at 1 individual/mL density in fish water at 28.5 °C on a 12 light:12 dark photoperiod. Larvae were not fed before or during the experimental period (from 7 to 8 days post fertilization (dpf)). All procedures were approved by the Institutional Animal Care and Use Committees at the CID-CSIC and conducted in accordance with the institutional guidelines under a license from the local government (agreement number 9027).

1.2. Zebrafish Monoamine-Oxidase (MAO) Activity

In this assay, 4-aminoantipyrine is oxidized and condensed with vanillic acid to produce a red quinoneimine dye. The assay was performed in a 96-well plate where 50 µL extracts were incubated in the presence of 100 µL of amine substrate tyramine, 10 mM final concentration and of 50 µL of a chromogenic solution containing final concentrations of 500 µM 4-aminoantipyrine, 1 mM vanillic acid and 4 U/mL horseradish peroxidase type II in 10 mM Phosphate Buffer pH 7.6. The reaction was left to stabilize for 15 min at room temperature and then incubated for a further 60 min at 28 °C in the microplate reader (Synergy 2, Bio Tek) where the formation of the red quinoneimine dye was recorded at 490 nm. MAO activity results were presented as nmol/min/mg obtained using the molar absorption coefficient for quinoneimine dye at pH 7.6 (4656 M^{-1} cm^{-1}) and normalized with total protein in assay.

1.3. Monoaminergic Neurotransmitters Extraction and Analysis

1.3.1. Chemicals and materials

Standards of dihydroxyphenylacetic acid (DOPAC), dopamine hydrochloride (DA), serotonin hydrochloride (5-HT) were purchased from Sigma-Aldrich (St. Louis, MO, USA); while 5-hydroxyindoleacetic acid (5-HIAA) was provided by Toronto Research Chemicals (TRC, Toronto, Canada), 3-methoxytyramine hydrochloride (3-MT) was obtained from Merck (Darmstadt, Germany), and norepinephrine (NE) was supplied by Tocris Bioscience (Ellisville, USA).

The labeled internal standards, 5-hydroxyindole-3-acetic acid d5, L-DOPA-2,5,6-d3 and L-Tyrosine-13C9,15N were all purchased from Toronto Research Chemicals (TRC, Toronto, Canada). Acetonitrile (ACN) HPLC LC-MS grade was supplied by VWR Chemicals (Leuven, Belgium) and ultra-pure water was obtained through Millipore Milli-Q purification system (Millipore, Bedford, USA). Formic acid (FA) was supplied by Fischer Scientific (Loughborough, UK) and ammonium formate by Sigma-Aldrich (St. Louis, MO, USA). Stock solutions of all the neurotransmitters (NTs) were prepared at 1000 ng μL^{-1} in MeOH, ultra-pure water or DMSO depending on their solubility. Calibration standards

were prepared in extractant solvent. A mix solution of all labelled standards (internal standard mixture, ISM) was prepared in extractant solvent (5 ng μL^{-1} for all of them, except from 5-HT which was 2 ng μL^{-1}). These standards were kept at −20 °C in amber vials to prevent degradation. The standards used for both calibration curve and QCs were freshly prepared every day in starting mobile phase.

1.3.2. Extraction

Monoaminergic neurotransmitters were extracted from heads of 8–9 pools of 20 larvae (8 dpf) following an extraction procedure adapted from Mayol et al. (2020). The extraction process was based on the use of a solvent of polarity sufficiently similar to the neurotransmitters to be able to extract them from the sample. Following the described process, 300 μL of cold extractant solvent (ACN:H2O (90:10) + 1% formic acid) were added to pools of 5 zebrafish larvae. Each sample was spiked with 10 μL of ISM (50 ng of each labeled NTs except for 5-HT-d4, 20 ng). Three stainless steel beads (3 mm diameter) were placed in each pool and samples were homogenized using a bead mill homogenizer (TissueLyser LT, Quiagen, Hilden, Germany). at 50 osc/min for 90 s. Subsequently, samples were shaken in a vibrating plate at 4 °C for 20 min and later centrifuged for 20 min at 13,000 rpm. Finally, the supernatant was filtered using 0.20 μm PTFE filters (DISMIC -13 JP, Advantec®) and kept at −80 °C until the analysis with LC-MS/MS. Temperature is a key element throughout the extraction process because NTs are very unstable and high temperatures can degrade these compounds.

1.3.3. LC-MS/MS Analysis

NTs profile was assessed by analyzing the extractions using ultra-high-performance liquid chromatography (Acquity UPLC® H-Class Waters, Milford, MA, USA) coupled to a triple quadrupole mass spectrometer equipped with an electrospray (ESI) source (Xevo, TQS micro,Waters, USA). To retain analytes, an Acquity UPLC BEH Amide column (150 mm × 2.1 mm ID, 1.7 μm particle size) provided with an Acquity UPLC BEH Amide precolumn (5 mm × 2.1 mm ID, 1.7 μm particle size) (Waters, Milford, MA, USA) was employed. The temperature was set at 30 °C. Mobile phase composition consisted of solvent A and solvent B. Solvent A was composed of Milli-Q water and acetonitrile (H2O:ACN) (95:5) containing 100 mM ammonium formate while solvent B was Milli-Q water and ACN (15:85) containing 30 mM ammonium formate. Both solvents were adjusted to pH 3 with FA. The LC gradient started at 100% B, decreased at 80% B in 4 min, and held for 1 min. From 5 to 7 min, B was linearly increased to 100%. Finally, initial conditions were re-equilibrated in 3 min. The total run time was 10 min. The flow rate was set at 250 μL min^{-1}. Samples were kept at 10 °C in the autosampler, and the injection volume was 10 μL. Regarding the MS conditions, desolvation gas flow was set to 900 L h^{-1} and the desolvation temperature was 350 °C. The cone gas flow was fixed at 150 L h^{-1}. Nitrogen was used as desolvation and cone gas., Source temperature was set at 100°C and a capillary voltage of 2.0 kV was applied. NTs were all measured under positive electrospray ionization (ESI+). The acquisition was performed in MRM mode. The optimum cone voltage (CV) and collision energies (CE) were taken from previous studies [1]. The first transition, corresponding to the most intense fragment, was used as the quantifier ion, whereas the second as the qualifier ion. The system and data management were processed using MassLynx v4.1 software package (Waters, Manchester, UK).

1.3.4. Quality Assurance

Calibration was performed over a concentration range from 0.005 to 2.5 ng μL^{-1}. The ISM was used as extraction and analytical quality control. 3-MT, DOPAC and 5-HIAA and were quantified by internal calibration, each with the respective labeled standard, while DA, 5-HT and NE were quantified using a external standard. Instrumental detection limits (IDLs) were determined using the lowest concentrated standard 0.005 ng μL^{-1} (except for LD at 0.010 ng μL^{-1}) that yielded a S/N ratio equal to 3. Method detection limits

(MDLs) were calculated using samples spiked at 50 ng. Intra-day precision was determined by two consecutive injections of 1 ng μL^{-1} standard solution and inter-day precision was determined by measuring the same standard solution for four different days. Moreover, recovery studies were performed with four replicates, using samples spiked at 50 ng with the neurotransmitter standard mixture and the ISM. In addition, matrix effect (ME) was assessed by comparing the peak area of each NT from the spiked sample (A) with the peak area of the analyte from the standard solution used in calibration curve (C) according to the equation: ME(%)=(A-B)/C × 100, where B is the peak area of each analyte from non-spiked samples (controls).

1.3.5. Quality Parameters

Great correlation coefficients (r2) were obtained over 0.99 for all analytes in a range from 0.005 to 2.5 ng μL^{-1} in most cases. Furthermore, IDLs were 0.46 pg (DOPAC) while MDLs varied from 1.7 (5-HT) to 57.8 ng larvae^{-1}. Intra-day precision ranged from 0.2% to 8.9% and inter-day precision values were from 2.6% to 13.2%. Regarding matrix effect, this parameter is an indicator of ionization suppression or enhancement of the analytes. Compounds with values below 70% indicated signal suppression due to the matrix (3-MT and DOPAC), whereas values above 130% suggested a signal enhancement.

2. Supplementary Results

Table S1. List of primers used for the qPCR.

Gene (Description)	ZFIN Acc Number	GenBank Acc Number		Sequence	Amplicon Length
mao (monoamine oxidase)	ZDB-GENE-040329-3	NM_212827.3	FW	5'-GCAGTCAGAGCCCGAATC	106 bp
			RV	5'-CACACCCATAAACTTGAG-GAATC	
tph1a (tryptophan hydroxilase 1a)	ZDB-GENE-030317-1	NM_178306.3	FW	5'-CAGTTCAGTCAGGAGATTGG	176 bp
			RV	5'-GACAGTGCGTGCTTCAG	
sert (sodium-dependent setotonin transporter *slc6a4a*)	ZDB-GENE-060314-1	NM_001039972.1	FW	5'-TAACCACTACAGTTTGGCTT-GATG	147 bp
			RV	5'-AACAGTTAACCGAGCTT-GTGAT	
vmat2 (vesicular monoamine transporter 2 *scl18a*2)	ZDB-GENE-080514-1	NM_001256225.2	FW	5'-TGGAGCTCTGCAGCTTTTT-GTGC	159 bp
			RV	5'-AACGCCGGCTCCAGCATAGC	
ppia2 (2-peptidylprolyl isomerase A)	ZDB-GENE-030131-8556	NM_212758.1	FW	5'-GGGTGGTAATGGAGCTGAGA	179 bp
			RV	5'-AATGGACTTGCCACCAGTTC	

Table S2. One-way ANOVA results of monoaminergic neurotransmitter levels.

	Description	*df* within Groups	*df* between Groups	F	Sig.
serotonin (5-HT)	monoamine neurotransmitter	5	29	40.579	<0.000
5-hydroxyindoleacetic acid (5-HIAA)	5-HT metabolite by MAO activity	5	29	3.283	0.018

norpinepherine (NE)	monoamine neurotransmitter synthesized from DA	5	29	7.506	<0.000
dopamine (DA)	monoamine neurotransmitter	5	29	6.489	<0.000
3,4- dihydroxyphenylacetic acid (DOPAC)	DA metabolite by MAO activity	5	29	9.789	<0.000
3-methoxytyramine (3-MT)	DA metabolite by COMT* activity	5	29	1.276	0.301

*catechol-O-methly transferase enzyme; *df*. Degrees of freedom; F. Fisher's coefficient; Sig. Significant levels (*P* value)

Table 3. Homogeneous Subsets for neurotransmitter levels following Tukey (HDS) post hoc analysis.

		N	Subset for alpha = 0.05			Group Letter
			**1**	**2**	**3**	
Serotonin	PCPA	5	27.908			a
	Fluoxetine + PCPA	6	29.777			a
	Fluoxetine	6	33.567			a
	Control	6	39.248			a
	Deprenyl + PCPA	6		63.670		b
	Deprenyl	6			110.663	c
	Sig.		0.617	1.000	1.000	
5-HIAA	PCPA	5	44.400			a
	Fluoxetine+PCPA	6	48.453	48.453		ab
	Fluoxetine	6	50.030	50.030		ab
	Deprenyl + PCPA	6	60.855	60.855		ab
	Deprenyl	6	61.087	61.087		ab
	Control	6		73.000		b
	Sig.		0.355	0.059		
Norepinephrine	PCPA	5	57.192			a
	Fluoxetine	6	76.078	76.078		ab
	Deprenyl + PCPA	6	95.977	95.977	95.977	abc
	Control	6		106.737	106.737	bc
	Fluoxetine + PCPA	6		113.573	113.573	bc
	Deprenyl	6			138.102	c
	Sig.		0.109	0.131	0.067	
Dopamine	PCPA	5	2517.306			a
	Control	6	3469.497			a
	Fluoxetine	6	3476.308			a
	Deprenyl + PCPA	6	3656.893			a
	Fluoxetine + PCPA	6	3786.715			a
	Deprenyl	6		5850.248		b
	Sig.		0.322	1.000		

DOPAC	Fluoxetine + PCPA	6	232.903		a
	Control	6	240.883		a
	Fluoxetine	6	364.188	364.188	ab
	PCPA	5		421.674	b
	Deprenyl	6		423.290	b
	Deprenyl + PCPA	6		444.845	b
	Sig.		0.052	0.444	
3-MT	Fluoxetine	6	207.158		a
	Fluoxetine + PCPA	6	208.647		a
	PCPA	5	217.432		a
	Control	6	217.975		a
	Deprenyl + PCPA	6	220.420		a
	Deprenyl	6	247.043		a
	Sig.		0.278		

Table S4. Homogeneous Subsets for gene expression results following Tukey (HDS) post hoc analysis. For *ppia2* CP (crossing point) values were used and ΔΔCT values were used for the remaining genes.

		N	**Subset for alpha = 0.05**			**Group Letter**
			**1**	**2**	**3**	
ppia2	Fluoxetine	8	17.200			a
	PCPA	8	17.354			a
	Deprenyl	8	17.600			a
	Control	8	17.810			a
	Deprenyl + PCPA	8	17.943			a
	Fluoxetine + PCPA	8	17.981			a
	Sig.		0.053			
tph1α	Deprenyl	8	−0.653			a
	PCPA	8	−0.409	−0.409		ab
	Deprenyl + PCPA	8	−0.293	−0.293	−0.293	abc
	Fluoxetine	8	−0.116	−0.116	−0.116	abc
	Control	8		0.000	0.000	bc
	Fluoxetine + PCPA	8			0.190	c
	Sig.		0.056	0.243	0.110	
mao	PCPA	8	−1.640			a
	Deprenyl + PCPA	8	−1.341			a
	Fluoxetine	8	−1.331			a
	Deprenyl	8	−1.127	−1.127		ab
	Fluoxetine + PCPA	8	−0.969	−0.969		ab
	Control	8		0.000		b
	Sig.		0.571	0.082		
vmat2	PCPA	8	−1.314			a
	Deprenyl	8	−1.130	−1.130		ab
	Deprenyl + PCPA	8	−1.046	−1.046		ab
	Fluoxetine	8	−0.699	−0.699		ab
	Fluoxetine + PCPA	8	−0.246	−0.246		ab
	Control	8		0.000		b
	Sig.		0.090	0.063		

Table S4. Continue of Table S4.

		N	Subset for alpha = 0.05			Group Letter
			1	2	3	
sert	Deprenyl	8	−1.025			a
	PCPA	8	−0.983			a
	Deprenyl + PCPA	8	−0.826			a
	Fluoxetine	8	−0.526			a
	Fluoxetine + PCPA	8	−0.044			a
	Control	8	0.000			a
	Sig.		0.354			
MAO Activity	Deprenly	6	−0.022			a
	Deprenly + PCPA	6	0.018			a
	Fluoxetine	6		0.438		b
	Control	6		0.445	0.445	bc
	PCPA	6		0.465	0.465	bc
	Fluoxetine + PCPA	6			0.495	c
	Sig.		0.217	0.637	0.068	

Table S5. Review of main observed results of this study. Arrows pointing up or down indicate significant increase or decrease of responses, respectively. Absence of responses are indicated by a hyphen.

Drug	Neurotransmitter Levels	Gene Expression	MAO Activity	Behaviour
5 µM Deprenyl	↑ 5-HT; DA and DOPAC	↓ *tph1a*; *mao* and *vmat2*	↓	↓ BLM; Stratle; VMR and Habituation
0.5 µM Fluoxetine	↓ 5-HIAA; ↑ DOPAC	↓ *mao*	—	↓ BLM; Stratle; VMR and Habituation
2.5 mM PCPA	↓ 5-HIAA; ↑ DOPAC	↓ *mao* and *vmat2*	—	↑ BLM and Stratle
5 µM Deprenyl + 2.5 mM PCPA	↑ 5-HT and DOPAC	↓ *mao* and *vmat2*	↓	↓ Habituation
0.5 µM Fluoxetine + 2.5 mM PCPA	↓ 5-HIAA	—	↑	↓ Habituation

Table S6. Animal Research: Reporting of Experiments (ARRIVE) guidelines checklist (doi:10.1371/journal.pbio.3000411).

1. Study Design	Control Group Is Included in this Study
	The experimental unit for neurotransmitters and MAO activity assessment is a pool of 20 larvae;<br>The experimental unit for behavior assessment is a single larva;<br>The experimental unit for gene expression assessment is a pool of 4 larvae.
**2. Sample Size**	The sample sizes used for each parameter has been indicated in the manuscript;<br>The sample sizes have been selected following previous extensive validations of the various used protocols. The number of animals was adapted to the endpoint analyzed, as the different endpoints exhibit different inter-individual variability (noise). We have a lot of experience analyzing all of these parameters in zebrafish and in the expected "signal" (difference between means of the groups) and noise for each endpoint. Therefore, the signal, the

	noise and a significance level of 0.05 were considered in the selection of the sampling size. With the selected sampling size, we were able to detect with precision differences with the control of about 30%. Whereas for most of the endpoints analyzed sampling size was 5-8, on the other hand, some behavioral analyses, known to have high intrinsic variability, sampling size was >100.
**3. Inclusion and Exclusion Criteria**	In behavioral analysis those animals were the tracking software failed or produced an error, or when more than one animal was added to each well, were excluded;<br>For the remaining analyses, no experimental unit was excluded.
**4. Randomization**	Randomization was included in all analyses. For behavior trails, experimental groups were randomly distributed throughout the microplates which were then randomly processed in the Danio Vision. For neurotransmitter, MAO activity and gene expression assessments, extractions and readings were conducted in the same day for all experimental groups, in a random order but always carefully taking notice that a representative from each experimental group was present in each subgroup.
**5. Blinding**	It was not possible to conduct blind strategy during experiments, behavior analyses, sample collection and MAO activity since it was conducted by the same person. However, blind strategy was implemented for gene expression and neurotransmitter assessment.
**6. Outcome Measures**	All outcome measures have been well defined in the manuscript.
**7. Statistical Methods**	Description of the implemented statistical methods, as well as the software used, are provided in the methods section 2.8. Parametric and non-parametric analyses was preformed according to data set normality. The type of analyses used for each set is identified in the figure caption.
**8. Experimental Animals**	All details of model species used is given in the manuscript
**9. Experimental Procedures**	Detailed experimental procedures have been included in the manuscript.
**10. Results**	Data sets are plotted as scatter plots with the median represented as a straight line. In the scatter plot, each point represents one experimental unit hence providing better visibility of data variability.

3. Supplementary References

1. Mayol-Cabré, M.; Prats, E.; Raldúa, D.; Gómez-Canela, C. Characterization of monoaminergic neurochemicals in the different brain regions of adult zebrafish. Sci. Total Environ. 2020, 745, 141205, doi:10.1016/j.scitotenv.2020.141205.
